# Predictors of Housing Trajectories Among Young Adults Experiencing Homelessness in Los Angeles

**DOI:** 10.1007/s11414-023-09863-2

**Published:** 2023-10-06

**Authors:** Eric R. Pedersen, Graham DiGuiseppi, Elizabeth J. D’Amico, Anthony Rodriguez, Denise D. Tran, Rupa Jose, Joan S. Tucker

**Affiliations:** 1 Keck School of Medicine, Department of Psychiatry and Behavioral Sciences, University of Southern California, 2250 Alcazar Street, Suite 2200, Los Angeles, CA 90033 USA; 2 School of Social Work, University of Southern California, Los Angeles, CA 90089 USA; 3 RAND Corporation, 4570 Fifth Ave #600, Pittsburgh, PA 15213 USA; 4 RAND Corporation, 1776 Main Street, PO Box 2138, Santa Monica, CA 90407 USA; 5 RAND Corporation, 20 Park Plaza # 920, Boston, MA 02116 USA

**Keywords:** Homeless, Youth, Young adult, Mental health, Substance use, Health

## Abstract

Experiencing homelessness during young adulthood is associated with negative health outcomes and understanding housing trajectories of young adults experiencing homelessness may aid in the development of evidence-based public health programs designed to serve this at-risk age group. In the present study, the authors examined baseline predictors of 24-month trajectories of housing stability and unsheltered housing among a sample of 271 young adults aged 18 to 25 recruited from drop-in centers in Los Angeles. In multivariate models, the authors found that identifying as multi-racial/other and better friendship quality at baseline were associated with less steep increases in the likelihood of stable housing over time. Being employed at baseline was associated with a less steep decrease in the probability of being unsheltered over time, while illicit drug use days associated with a steeper decrease in the probability of being unsheltered over time. Continued research is needed to establish important factors determining young adults’ long-term housing trajectories in the effort to promote greater access and engagement with housing services.

## Introduction

Homelessness has adverse consequences on the health and development of young people, and young adults who experience homelessness are a large and understudied population who are often marginalized. Nearly 3.5 million young adults (ages 18–24 years) in the USA experience homelessness annually,^1^ with over 27,000 young adults in the USA experiencing homelessness on a given night.^2^ Experiencing homelessness during young adulthood is associated with both immediate and long-term negative health and psychological outcomes, including behavioral health problems (e.g., posttraumatic stress disorder, depression), physical health complications, and heavy use of alcohol, cannabis, and illicit drugs.^3–7^ These physical and behavioral health problems serve as both determinants and consequences of other challenges faced by young people who experience homelessness, including physical and sexual violence, risky sexual behaviors, and legal difficulties.^8–11^ Young adults who experience homelessness are also more likely to experience early mortality compared to those who are more stably housed.^12^

Understanding factors associated with both stable and unstable housing among young adults experiencing homelessness is a necessary first step towards designing programs and policies to reduce the negative physical and mental health effects. The experience of homelessness is a heterogenous one,^13^ making longitudinal studies outlining the course of housing and its determinants crucial in providing a more in-depth understanding of these issues. Existent longitudinal studies have examined housing trajectories of young people experiencing homelessness. For example, Milburn and colleagues^14^ examined 183 adolescents experiencing homelessness in Los Angeles and found that social and familial support (i.e., engagement with prosocial peers, maternal support) and attendance in school predicted stable housing outcomes over 2 years, whereas exposure to family violence and reliance on use of shelter services predicted less stable housing. Braciszewski and colleagues^15^ found in a 7-year longitudinal study of 243 adolescents that previous homelessness, racial/ethnic minority status, and neighborhood income were related to more difficulty securing stable housing. In another study of 359 Canadian young adults experiencing homelessness, Roy and colleagues^16^ found that factors related to social integration (e.g., having a high school degree, seeking psychological services) were associated with housing stability over 90 days, whereas factors related to street entrenchment (e.g., injection drug use, informal income sources such as from selling drugs or panhandling) were associated with housing instability. Other studies assessing mixed samples of both adolescents and young adults in the USA have found that risk factors for housing instability included illicit drug use and engagement in high-risk sexual behaviors, whereas strong social support networks, younger age, less time homeless, having the ability to return home if needed (e.g., not forced out of their home by parents), and receipt of behavioral health services were all protective.^17–20^

Though the available studies help with our understanding of trajectories of homelessness among young people, most focus on adolescents or have samples comprised of both adolescents and young adults. Young adults are in a crucial developmental period, where they start experiencing more autonomy from parents and begin developing their own identity, including establishing financial independence and fostering intimate relationships. It is also a time, after the age of 18, when there are less protections from local, state, and federal government, making options for housing and educational opportunities more limited for young adults experiencing homelessness. Experiencing homelessness during this age period can have significant implications for future outcomes,^21–23^ and thus, an increased understanding of housing trajectories of young adults experiencing homelessness in the USA may aid in the development of evidence-based public health programs and policies designed to serve this age group. Moreover, though research has established that mental, physical, and social health outcomes are affected by homelessness, it is less clear how these factors are associated with prospective housing trajectories among young adults. Moreover, young adults’ experiences of homelessness are often heterogeneous,^13^ with some young adults spending periods of time in their own home, interspersed with temporary housing accommodations with family, friends, or strangers (“couch surfing”); in shelters; or in highly unstable and dangerous unsheltered settings. Thus, in addition to examining stable housing as an optimal outcome, studies need to examine predictors of unsheltered housing trajectories, which encompass living in outdoor locations, vehicles, or abandoned buildings. Such experiences can be particularly detrimental for young adults, even if experienced temporarily.^24, 25^

The present study addresses gaps in the literature by examining predictors of trajectories of housing stability and unsheltered housing, grouped into four key areas pertinent to young adults experiencing homelessness: (1) demographics: age, birth sex, sexual and gender minority identification, race and ethnicity, and age of first homeless experience; (2) substance use behaviors: heavy drinking days, cannabis use days, and days of illicit drug use; (3) health and social functioning; and (4) use of housing services. Establishing the association between these factors (measured at a single point-in-time) and prospective housing trajectories over 2 years is crucial to advancing efforts to support the housing needs of these young adults.

## Methods

### Participants and procedures

Participants were part of an evaluation of AWARE, a brief substance use and sexual risk reduction program for 18 to 25 year olds experiencing homelessness. Findings from the intervention trial and description of the intervention can be found elsewhere.^26, 27^ Data for the present analyses come from the baseline survey and from four follow-up surveys (3, 6, 12, and 24 months post-baseline). Participants were recruited from three drop-in centers serving young adults experiencing homelessness in Los Angeles County. Drop-in centers provide services to address the basic needs of young people experiencing homelessness (food, clothing), but oftentimes offer higher level services such as case management and other programs to meet health and social service needs. The three drop-in centers included in this study were diverse in location (e.g., Hollywood, Venice/Santa Monica) and population served, with one of the two drop-in centers in Hollywood offering services specifically for sexual and gender minority youth experiencing homelessness.

To be eligible for the study, participants needed to (1) be between the ages of 18 and 25, (2) be currently seeking any services at one of the drop-in centers, (3) plan to be in the study area for the next month, (4) be willing to provide contact information for follow-up surveys, (5) be reachable by e-mail or phone for follow-up, (6) be English-speaking, and (7) display no evidence of cognitive impairment at screening. All procedures were approved by the institution’s Internal Review Board.

Three hundred and seventy-one young adults at the drop-in centers were approached for screening, resulting in a final sample of 276 participants (see Tucker et al.^22^ for more details). Five participants had missing data on baseline predictor variables, resulting in an analytic sample of 271. Table 1 contains a description of the sample, which was about 22 years old on average, mostly male sex at birth (72%), and non-White (84%), with 45% reporting sexual and gender minority identity (42.7% reported sexual minority identification and 13.4% reported gender minority identification, with 28% of sexual minorities and 86% of gender minorities also identifying with the other minority identification). Sample demographics were similar to the demographic profile of the population of young people experiencing homelessness in Los Angeles County.

**Table 1:** Participant characteristics at baseline. Note: ^1^Responses of gay, lesbian, bisexual, questioning, or asexual for the sexual orientation survey item. Four participants did not respond to the sexual orientation item. ^2^Responses of “gender neutral,” “other gender,” or “transgender” to the gender identification item. ^3^Use of housing services value corresponds with a response option of use of housing services “3 to 5 days” in the past 3 months

Variable	Mean (SD)	Percentage
*Demographics/control variables*		
Age	22.1 (1.8)	--
Female birth sex	--	27.5%
Sexual/gender minority	--	44.9%
Sexual minority^1^		42.7%
Gender minority^2^		13.4%
Race and ethnicity		
Black	--	36.7%
Hispanic/Latinx	--	29.8%
White	--	16.4%
Multi-racial/other	--	17.1%
Age first homeless	17.3 (2.6)	--
Drop-in center location		
Hollywood drop-in #1	--	28.3%
Hollywood drop-in #2	--	37.7%
Venice drop-in	--	34.1%
Received AWARE intervention	--	47.8%
*Substance use (past 30 days)*		
Number of heavy drinking days	2.7 (5.8)	--
Number of cannabis use days	17.4 (13.2)	--
Number of illicit drug use days	3.0 (6.9)	--
*Health and Social Functioning*		
General health	2.3 (1.2)	--
Depression	--	30.1%
Friend relationship quality	3.0 (1.3)	
Pregnancy (self or someone else)	--	3.6%
In school	--	26.1%
Employed	--	22.6%
*Service use*		
Use of housing services^3^	3.2 (2.1)	--

Baseline surveys were completed in person via paper-pencil survey, whereas follow-up surveys were generally complete via online survey or phone interview. Our team has extensive experience tracking young people who experience homelessness and have developed tracking and locator information to limit attrition.^28^ Thus, 87% of the sample was retained at the 24-month follow-up.

### Measures

#### Demographics and control variables

Participants’ age, birth sex (male or female), gender identification, sexual orientation, race, and ethnicity were assessed. A dichotomous variable indicated “Sexual / Gender minority” was created and set equal to 1 if participants reported a gender identity that was different from their birth sex, transgender identity, or non-heterosexual orientation (this variable was equal to 0 if participants’ gender identity matched their birth sex and if participants reported “straight/heterosexual” orientation). Age at first homelessness was assessed with the question “How old were you the first time you left home and were living on your own, apart from a parent or guardian [even if it was just a short period of time]?” Being currently enrolled in school full- or part-time (vs. not in school) and currently employed full- or part-time (vs. unemployed) were also assessed. Dummy variables for drop-in center location (with the Venice drop-in center as reference) and intervention group (1 = AWARE intervention, 0 = usual care at drop-in) were included as control variables and effects were not interpreted.

#### Substance use

Use of three types of substances in the past 30 days was assessed. Heavy alcohol use was assessed by first presenting participants with a definition and images of standard drinks (i.e., “one regular size can/bottle of beer or wine cooler, one 5 ounce glass of wine, one mixed drink, or one shot glass of 1.5 ounce liquor”). Heavy alcohol use, defined according to the National Institute on Alcohol Abuse and Alcoholism, was assessed as the number of days participants reported drinking five or more drinks of alcohol in a row “within a couple of hours.” Past 30-day use of 13 classes of drugs (e.g., cannabis, methamphetamine, prescription drug misuse) was also assessed. Number of cannabis use days was the number of days participants reported using “marijuana or hashish.” Number of illicit drug use days was assessed by asking “How many days did you use any of the drugs listed above, not including marijuana?”

#### Health and social functioning

Six variables were used to describe participants’ health and social functioning. Participants reported their general health, ranging from “Excellent” (1) to “Poor” (5). Depression symptoms in the past 2 weeks were assessed using the eight-item Patient Health Questionnaire [PHQ-8;^29^]. Probable depression diagnosis (1 = yes, 0 = no) was indicated by a PHQ-8 score of greater than or equal to 10. Friendship relationship quality was assessed using the PROMIS Pediatric Peer Relationships Scale^30^ which consists of the mean of three items (e.g., “I was able to count on my friends”) with response options ranging from “Never” (1) to “Almost Always” (5). A binary variable also indicated if participants had been pregnant or had impregnated someone else in the past three months (1 = yes, 0 = no).

#### Use of housing services

Participants were asked how often they used formal services “at a drop-in center or other agency/organization” in the past 3 months. Participants indicated the number of days they used services “to help you find housing,” with response options on a six-point ordinal scale ranging from “0 days” (1) to “more than 15 days” (6).

#### Housing

At each survey, participants’ housing situation was assessed with the item: “In the past 3 months, on average, how often have you spent the night in each of the following places?” This was followed by a list of 10 different housing options, with eight response options for each choice ranging from “Never” to “Every day.” From these items, two dichotomous outcome variables were created. Participants were considered *stably housed in their own home* if they selected “Every day” for the item “Your own house, apartment or room” and received a value of 1 on this outcome variable. Participants who chose any option less than “Every day” (e.g., “Never” to “4-5 times a week”) were considered not stably housed and received a value of zero on this outcome variable. Options for housing that were not considered stably housed in their own home were staying temporarily in someone else’s apartment or house, in an emergency shelter or transitional housing program, outdoors or on the street, in a car or vehicle, in an abandoned building, in a hotel or motel, or somewhere else temporarily. Secondly, *being unsheltered* was indicated (outcome variable = 1) if participants reported that they had spent at least one night (i.e., “Less than once a month” to “Every day”) in at least one of the following places in the past 3 months: “Outdoors, the street, or a park,” “Car or other private vehicle (e.g., van, camper),” or “Abandoned building.” Participants who reported “Never” staying in all three of these places received a value of zero on this outcome variable.

### Analytic plan

Latent growth curve modeling was used to estimate trajectories of stable housing and being unsheltered. Separate growth models were fit for each outcome. Because both outcomes were dichotomous, the authors used a maximum likelihood estimator and logit link. Model specification for categorical outcomes followed a common method,^31^ in which means of the intercepts were fixed at zero, and slopes were freely estimated. Different models were specified for no growth, linear, and quadratic change trajectories to determine the best fitting and most appropriate unconditional model. After arriving at the best fitting unconditional model, growth factors (i.e., intercepts and slopes) were regressed on covariates (demographics, substance use, health and social functioning, and service use). The authors undertook a model building process in which covariates were added as predictors of slope factors one at a time in a series of separate (bivariate) models. Covariates that were associated with each slope factor at *p* ≤ 0.10 in bivariate models were then included in a final multivariate model. All regression coefficients are presented as standardized parameter estimates with accompanying standard errors. Missing data were minimal across baseline variables (*n* = 1 missing for race/ethnicity and age; *n* = 2 missing for health, employment, and use of housing services). Therefore, the default in Mplus for handling missing data was used, in which cases with missing data on predictor variables were excluded from multivariate models.

## Results

### Trajectory and predictors of stable housing

**Figure 1:**
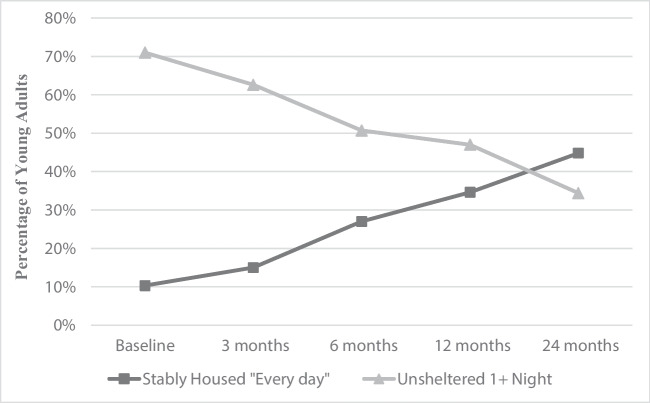
Observed trajectories of participants’ housing in the past 3 months

Specifying the unconditional latent growth curve model for stable housing, a linear model was a better fit than the no growth model, as indicated by significant improvements in negative two log likelihood (Δ −2LL = 104.9, *p* < 0.001) and AIC and BIC values. A quadratic model improved model fit further (Δ −2LL = 22.8, *p* < 0.001), but the linear model was chosen as it was more parsimonious and better allowed for estimation of the effects of covariates. The model estimated a 14.5% probability of being in one’s own home at baseline (observed: 10.3%), with significant variance in the intercept (*σ* = 2.16, *SE* = 0.71, *p* = 0.002). There was a significant mean increase in the likelihood of being in one’s own home every night over the course of the study (*μ*_slope_ = 0.29, *SE* = 0.04, *p* < 0.001), with a non-significant variance in the slope factor (*σ* = 0.04, *SE* = 0.04, *p* = 0.29). The estimated probability of being in one’s own home at the 24-month follow-up was 48.2% (observed: 44.7%) (see Fig. 1 for observed proportions of being stably housed over time).

Associations between covariates and stable housing growth factors are shown in Table 2. In bivariate models, female birth sex, race and ethnicity, being in school, being employed, heavy drinking days, friend relationship quality, and use of housing services were associated (*p* ≤ 0.10) with intercept or slope factors. In the multivariate model, female birth sex (*β* = 0.40, *p* = 0.04), and friend relationship quality (*β* = 0.30, *p* = 0.01) were associated with a greater likelihood of stable housing at baseline. Furthermore, identifying as multi-racial/other (compared to being White) (*β* = −1.35, *p* = 0.03) and friend relationship quality at baseline (*β* = −0.66, *p* = 0.001) were associated with less steep increases in the likelihood of stable housing over time.

**Table 2:** Predictors of stable housing over 2 years (*N* = 271). Note. ^a^*p* < 0.10 *p < 0.05; models controlled for intervention group and drop-in center site

Variable	Bivariate regressions		Multivariate model	
	Intercept	Linear	Intercept	Linear
	*β* (SE)	*β* (SE)	*β* (SE)	*β* (SE)
*Demographics/control variables*				
Age	−0.01 (0.11)	0.13 (0.17)		
Female birth sex	0.45 (0.23)*	0.03 (0.39)	0.40 (0.19)*	
Sexual/gender minority	0.11 (0.22)	0.32 (0.34)		
Black (vs. White)	0.47 (0.32)	−1.01 (0.55)^a^		−0.84 (0.55)
Hispanic/Latinx (vs. White)	0.06 (0.34)	−0.38 (0.51)		−0.56 (0.58)
Multi-racial/other (vs. White)	−0.19 (0.40)	−0.75 (0.59)		−1.35 (0.62)*
Age first homeless	−0.16 (0.11)	0.29 (0.18)		
*Substance use*				
Heavy drinking days	0.06 (0.11)	−0.30 (0.18)^a^		−0.30 (0.18)
Cannabis use days	−0.02 (0.11)	0.11 (0.17)		
Illicit drug use days	−0.03 (0.02)	−0.01 (0.03)		
*Health and social functioning*				
General health	−0.19 (0.11)	0.19 (0.19)		
Depression	−0.14 (0.24)	0.29 (0.38)		
Friend relationship quality	0.31 (0.11)*	−0.96 (0.18)*	0.30 (0.11)*	−0.66 (0.19)*
Pregnancy (self or someone else)	0.14 (0.56)	−0.70 (0.86)		
In school	0.52 (0.23)*	−0.31 (0.40)	0.19 (0.21)	
Employed	0.43 (0.24)^a^	−0.20 (0.39)	0.32 (0.20)	
*Service use*				
Use of housing services	−0.23 (0.11)*	0.21 (0.18)	−0.15 (0.10)	

### Trajectory and predictors of being unsheltered

A linear growth model of being unsheltered on at least one night in the past 3 months was a better fit than the no growth model (Δ −2LL = 88.2, *p* < 0.001), and the quadratic growth model was a better fit than the linear model (Δ −2LL = 15.02, *p* = 0.02). However, fit of the linear growth model was already quite good (*χ*2 = 17.23, *df* = 26, *p* = 0.90), and the linear model was thus chosen for parsimony (see Fig. 1 for observed proportions of being unsheltered over time). The linear growth model estimated a 65.8% probability of being unsheltered at baseline (observed = 69.5%), and significant mean decreases in the probability of being unsheltered over time (*μ*_slope_ = −0.27, *SE* = 0.04, *p* < 0.001). The estimated probability of being unsheltered at the 24-month follow-up was 32.8% (observed = 34.4%). There was significant variance in the intercept (*σ* = 2.94, *SE* = 0.80, *p* < 0.001), and marginally significant variance in the slope factor (*σ* = 0.06, *SE* = 0.04, *p* = 0.10). The covariance between the intercept and slope was not significant (cov. = −0.18, *p* = 0.12), suggesting no significant association between the probability of being unsheltered at baseline and the probability of change over time.

Associations between covariates and growth factors for being unsheltered are displayed in Table 3. In bivariate models, reporting sexual or gender minority identity, being in school, being employed, number of cannabis use and illicit drug use days, and friend relationship quality were associated with intercept or slope factors (*p* ≤ 0.10). When including these variables in the multivariate model and interpreting the significant effects, being in school (*β* = −0.36, *p* = 0.03) and being employed (*β* = −0.58, *p* = 0.003) were associated with a lower probability of being unsheltered at baseline, and reporting more illicit drug use days was associated with a greater probability of being unsheltered at baseline (*β* = 0.34, *p* < 0.001). Furthermore, being employed at baseline was associated with a less steep *decrease* in the probability of being unsheltered over time (*β* = 0.61, *p* = 0.03), while illicit drug use days was associated with a steeper decrease in the probability of being unsheltered over time (*β* = −0.34, *p* = 0.03).

**Table 3:** Predictors of being unsheltered over 2 years. Note. ^a^*p* < 0.10 *p < 0.05; models controlled for intervention group and drop-in center site

Variable	Bivariate regressions		Multivariate model	
	Intercept	Linear	Intercept	Linear
	*β* (SE)	*β* (SE)	*β* (SE)	*β* (SE)
*Demographics/control variables*				
Age	0.04 (0.09)	−0.03 (0.13)		
Female birth sex	−0.17 (0.20)	−0.13 (0.30)		
Sexual/gender minority	−0.26 (0.30)	−0.12 (0.07)^a^		−0.31 (0.22)
Black (vs. White)	−0.14 (0.26)	0.58 (0.41)		
Hispanic/Latinx (vs. White)	0.06 (0.28)	0.40 (0.42)		
Multi-racial/other (vs. White)	0.29 (0.32)	0.72 (0.47)		
Age first homeless	−0.04 (0.09)	−0.16 (0.13)		
*Substance use*				
Heavy drinking days	0.07 (0.09)	0.05 (0.12)		
Cannabis use days	−0.09 (0.09)	0.28 (0.13)*		0.19 (0.11)^a^
Illicit drug use days	0.34 (0.10)*	−0.30 (0.16)^a^	0.34 (0.10)*	−0.34 (0.15)*
*Health and social functioning*				
General health	0.11 (0.09)	−0.15 (0.14)		
Depression	0.01 (0.20)	0.22 (0.29)		
Friend relationship quality	−0.23 (0.09)*	0.17 (0.14)	−0.12 (0.07)	
Pregnancy (self or someone else)	0.35 (0.49)	−1.04 (0.76)		
In school	−0.63 (0.19)*	0.32 (0.31)	−0.36 (0.17)*	
Employed	−0.64 (0.20)*	0.69 (0.32)*	−0.58 (0.20)*	0.61 (0.29)*
*Service use*				
Use of housing services	0.08 (0.09)	−0.18 (0.13)		

## Discussion

The current study examined 2-year housing trajectories in a sample of 271 young adults initially experiencing homelessness in Los Angeles, focused on housing stability (i.e., living in one’s own home every day) and being unsheltered (i.e., spending at least one night on the street, in a vehicle, or in an abandoned building) in the past 3 months. Regarding housing stability, several factors associated with trajectories of young adults report of living in their own home every day for the past 3 months. After accounting for other factors significant in bivariate models, both female birth sex and better quality of one’s friendships were associated with a greater likelihood of being stably housed at baseline, and reporting multi-racial/other race/ethnic identification (compared to White) and better friendship quality were associated with a less pronounced increase in the probability of being stably housed over time. Findings suggested that targeted outreach efforts to provide stable housing interventions for male young adults and those identifying with multiple racial/ethnic identities may be necessary. As in prior work, social support (examined in this study as self-reported quality of friendships) was associated with stable housing cross-sectionally at baseline.^17, 19, 20^ Strong peer relationships have been associated with increased use of drop-in centers and higher-level services.^9, 32, 33^ and also reduce the mental and physical health consequences of homelessness as young people have friends to rely on for a place to stay or to help them in times of mental or physical health needs.^34, 35^ To our surprise, however, greater social support at baseline was associated with a lower probability of stable housing over time. It is possible that stronger friend networks may help address young adults’ housing needs for a brief period of time (i.e., “couch surfing”), but staying with friends is inherently an unstable long-term housing situation. It is also possible that young adults experiencing homelessness develop strong relationships within “street families,”^36^ which can mimic traditional family roles and be difficult to give up if transitioning to a more stable housing setting. Entrenchment in street life has also been associated with less housing stability in other work.^16^ Thus, to encourage obtaining more stable housing, interventions that target young adults more fully engrained in street life may be necessary.

Regarding trajectories of being unsheltered for at least one night in the past 3 months, several factors were significant at the bivariate level (reporting sexual and gender minority identity, being in school, being employed, cannabis and illicit drug use, and friendship quality). However, when all variables were included in the model, only being in school and being employed were associated with a lower probability of being unsheltered at baseline, and being employed was associated with a less steep *decrease* in the probability of being unsheltered over time. This is contrary to other work, which showed that engagement in school and work may be important protective factors against being unsheltered, a particularly high-risk living situation.^1, 37, 38^ It should be noted that we did not assess forms of employment, so it is unclear if reported employment was formal/steady jobs with a paycheck or more “off-the-books” types of employment. Frequency of illicit drug use was associated with a greater probability of being unsheltered at baseline, consistent with prior research showing that drugs besides alcohol and cannabis are typically associated with greater risk of being unsheltered among young adults experiencing homelessness.^18, 39^ Interestingly, greater illicit drug use at baseline was associated with a more pronounced decrease in the probability of being unsheltered over time, potentially indicating that young adults reporting more problematic substance use could have been receiving targeted housing services (shelter accommodations or permanent supportive housing) or been prioritized for receiving alcohol and other drug services due to severity.

Results should be considered with several limitations in mind. First, our sample was limited to young adults in the Los Angeles area, and although the sample was diverse and reflected the broader population of homeless young adults in Los Angeles, findings may not be generalizable to populations outside of Los Angeles. In addition, all data, including reports of pregnancy and illicit drug use, were based on self-report, which has potential for respondent bias. Sexual and gender minority participants were included as one group for analyses, and small sample sizes within each specific identity (e.g., transgender or gender non-binary participants, lesbian cisgender women or gay men) did not allow for meaningful examination of trajectories by these unique groups. Lastly, though relevant literature was reviewed to inform selection of factors associated with prospective housing trajectories in our analyses, there are other factors that may affect housing which were not included, such as exposure to family violence and neighborhood/environmental level factors. More research is needed on both risk and protective factors that may contribute to stable housing to provide a better understanding of the services needed to alleviate the burden of homelessness among young people.

## Conclusion

In conclusion, this study fills an important gap in the literature on young adults experiencing homelessness. Though studies exist, there are few longitudinal studies of young adult housing outcomes. It is important to continue to understand factors associated with both stable and unstable housing among young adults experiencing homeless in the effort to develop programs and policies to reduce homelessness and its negative physical and mental health effects on young people.

## Implications for Behavioral Health

Though it was not surprising that use of housing services was negatively associated with stable housing at baseline (those with stable housing tended to use housing services *less* because they did not need the services), use of housing services at baseline was not associated with trajectories of housing stability or being unsheltered over time. Housing services are often not a primary reason for young adults’ use of drop-in center services,^32^ because these centers tend to focus on providing basic needs services (food, showers); this is also shown in our drop-in center sample here, with participants using housing services on an average of 3 to 5 days in the past 3 months at baseline. Helping engage youth in drop-in center services is a necessary first step towards connecting young adults to services that address their housing and health needs. In addition, none of the physical or mental health factors (e.g., depression, general health) was associated with either stable housing or unsheltered housing trajectories. It is possible that because these young adults were recruited from drop-in centers, they were more likely to have their health needs met (e.g., physical health was rated an average of “very good”), and perhaps those with depression (30% met criteria in our sample) were already receiving some services at the drop-in for symptoms.^40, 41^ Still, poor mental and physical health are clearly linked to homelessness,^42^ and continued outreach, assessment, and intervention are necessary to meet young adults’ needs.

## Data Availability

Data is available from the corresponding author by reasonable request.
